# ERrrr…Where are the Progenitors? Hormone Receptors and Mammary Cell Heterogeneity

**DOI:** 10.1007/s10911-015-9336-1

**Published:** 2015-07-21

**Authors:** Giusy Tornillo, Matthew J. Smalley

**Affiliations:** European Cancer Stem Cell Research Institute, School of Biosciences, Cardiff, CF24 4HQ UK

**Keywords:** Hormone receptor, Mammary epithelium, Cell heterogeneity, Stem cell, Progenitor cell, Oestrogen, Progesterone

## Abstract

The mammary epithelium is a highly heterogenous and dynamic tissue that includes a range of cell types with varying levels of proliferative capacity and differentiation potential, from stem to committed progenitor and mature cells. Generation of mature cells through expansion and specification of immature precursors is driven by hormonal and local stimuli. Intriguingly, although circulating hormones can be directly sensed only by a subset of mammary cells, they also regulate the behaviour of cells lacking their cognate receptors through paracrine mechanisms. Thus, mapping the hormonal signalling network on to the emerging mammary cell hierarchy appears to be a difficult task. Nevertheless, a first step towards a better understanding is the characterization of the hormone receptor expression pattern across individual cell types in the mammary epithelium. Here we review the most relevant findings on the cellular distribution of hormone receptors in the mammary gland, taking into account differences between mice and humans, the methods employed to assess receptor expression as well as the variety of approaches used to resolve the mammary cell heterogeneity.

## Introduction

Systemic hormones are crucial regulators of mammary gland development and function throughout reproductive life [1, 2]. Oestrogen and growth hormone drive ductal elongation at the onset of puberty, whereas cyclic fluctuations of oestrogen and progesterone sustain subsequent remodelling of the ductal network [1, 3]. During pregnancy, progesterone and prolactin induce the formation of alveolar structures capable of milk production [1, 4].

Although the morphogenetic responses elicited by reproductive hormones in the mammary gland have been extensively studied for decades, the precise mechanisms of hormone action and the underlying cell dynamics have only recently emerged. It is now well established that in the mammary epithelium a subset of cells can directly sense systemic hormones and convert them into local signals to influence the behaviour of neighbouring cells [2, 5]. However, the discovery of a hierarchical cell organization in the mammary epithelium has further complicated this scenario, prompting the investigation of the hormone receptor status across various mammary cell types with distinctive proliferative capacities and differentiation potentials. Mapping hormone receptor expression along the mammary cell hierarchy is crucial for understanding how hormonal signalling coordinates the recruitment and lineage commitment of stem/progenitor cells in support of normal mammary tissue homeostasis and remodelling. Moreover, deciphering this puzzle may reveal how disruption of hormone receptor patterning can contribute to development and maintenance of breast cancer.

This review summarizes our current knowledge regarding the cellular distribution of hormone receptors in the mouse and human mammary epithelium, with a primary focus on the expression pattern of the receptors for the ovarian steroid hormones oestrogen and progesterone (ER and PR, respectively). Furthermore, given the presence of heterogeneity in the hormone receptor-positive population, we will also discuss recent studies, which underscore that not all the hormone receptor-positive cells are equivalent with respect to their fate and function in the mammary epithelium.

## Cell Heterogeneity and Hormone Receptors in the Mammary Epithelium: Biology and Methodology at a Glance

The mammary epithelium consists of an outer layer of basal cells, most of which are contractile myoepithelial cells, and an inner layer of luminal cells [6]. Luminal cells include ductal and alveolar epithelial cells, which line the ducts and form the alveoli that develop during pregnancy, respectively. Luminal alveolar cells primarily function as milk-secreting cells throughout lactation [6]. These cell lineages derive from undifferentiated precursors, which expand and progress along specific differentiation pathways in response to hormonal and local stimuli [6]. Of course, the different stages through which the mammary epithelium develops have different hormonal contexts; these ultimately determine the extent of progression towards terminal differentiation along any lineage pathway as well as the degree of expansion in cell number within any lineage required to fulfil the functional purpose of the tissue.

Over the past recent years, our understanding of the mammary hierarchical cell organization has significantly increased. Combining fluorescence-activated cell sorting (FACS) and a variety of functional assays, epithelial cell populations with features of stem, progenitors and mature cells have been identified and characterized both in the mouse and human mammary gland [7–9]. In spite of some differences in the panels of cell surface markers between species, it seems that analogous subpopulations have highly conserved gene expression profiles and functions [7–9].

Based on this cell separation / functional analysis approach, stem cells have been localised to the basal cell layer, whereas progenitors have been largely found in the luminal compartment [7–9]. Mouse mammary stem cells (MaSCs) have also been referred as mammary repopulating units (MRUs), due to their ability to regenerate the complete mammary epithelium when injected into a mammary fat pad cleared of the endogenous epithelium [10, 11]. Similarly, human MRUs can be identified upon transplantation under the renal capsule of immunodeficient mice or into “humanized” mouse mammary fat pads [10, 11]. Additionally, MaSCs are recognized for their ability to resist from anoikis in vitro in non-adherent cultures and generate floating colonies, known as mammospheres [11]. In contrast, by virtue of their high proliferative potential but limited repopulating capacity, progenitor cells are detectable in vitro by colony-forming cell (CFC) assays [10]. Terminally differentiated cells do not transplant in vivo or form colonies in vitro and appear unable to proliferate, at least in the current tested conditions.

The use of dissociated mammary epithelial cells, even when freshly isolated, can be an important caveat to MRU and CFC assays in determining cell regenerative and differentiation potential. In fact, it is now emerging that the plasticity of mammary cells, observed after tissue disaggregation and loss of the original homeostatic context, may not reflect behaviour of cells in intact glands. Unlike such assays, cell lineage tracing experiments allow to track stem and progenitor cells and their progeny in situ*.* Yet lineage tracing has highlighted the possible existence of bipotent stem cells as well as long-lived unipotent cells and a variety of precursors that are recruited during morphogenesis and homeostasis in the mammary gland [7, 12–16]. However, discrepancies among results from separate studies indicate that caution is necessary during the design and analysis of transgenic reporter models for lineage tracing.

In contrast to the doubt that still remains over how many ‘stem-like’ cell populations exist and their location in the mammary epithelium, cells which express the intracellular receptors for the steroid hormones oestrogen and progesterone have been well-studied in situ. Multiple and functionally distinct isoforms of the oestrogen receptor (ER) and the progesterone receptor (PR) are found in mammary cells [17] and, as a result of their dimerization, the functional variety of these receptors is likely to be wider, including various ER homo- and hetero-dimers as well as various PR homo- and hetero-dimers.

There are two major ER isoforms, ERα and ERβ. Both in rodents and in humans ERα is confined to the luminal layer of the mammary epithelium [18–20], whereas ERβ has a widespread distribution [21, 22]. The two main PR forms, PRA and PRB, largely co-localize and are both restricted to the luminal epithelium in human breast [23]. However, differential expression of these two isoforms has been observed at distinct stages of development in the mouse mammary gland, with the presence of only PRA in virgin animals and the preferential expression of PRB during pregnancy [24]. As most of the studies reviewed here are related to ERα and do not distinguish the two PR forms, hereafter ER will refer to ERα while PR will not refer exclusively to PRA or PRB, unless otherwise specified.

**Fig. 1:**
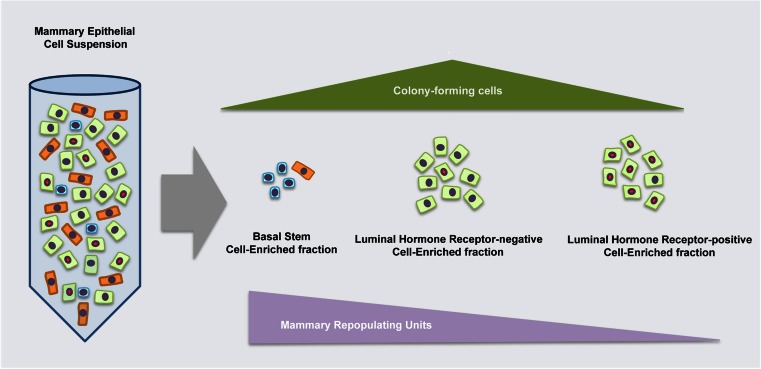
Properties of distinct mammary epithelial cell populations. Based on the expression of specific cell surface markers, mammary epithelial cells can be fractionated by FACS in basal, luminal hormone receptor-positive and luminal hormone receptor-negative cells. The fraction enriched in hormone receptor-positive cells contains stem and progenitor cells at very low frequency, as assessed by transplantation experiments and in vitro colony forming assays, respectively

In situ analysis of ER and PR expression has demonstrated a precise spatial distribution of the cells that can be directly targeted by steroid hormones in the mammary gland [18–20, 23, 24], but provided limited information about the functional properties of these cells. In order to define the position of hormone sensing cells along the mammary differentiation hierarchy, mammary biologists have prospectively isolated discrete putative stem, progenitor and mature cells by flow cytometry and analyzed them for the expression of steroid hormone receptors [25–35] (Tables 1 and 2). Alternatively, they have employed cell surface markers to enrich for steroid hormone receptor-positive or negative cells and assessed their growth and differentiation potential using in vitro and in vivo assays [29–32] (Fig. 1). Gene expression analysis on these isolated cell fractions has revealed the average ER and PR transcript levels for each population, whereas analysis of ER and PR staining in single sorted cells has provided mainly qualitative information on the heterogeneity of the hormone receptor status within distinct populations. Importantly, the intrinsic sensitivity of the method employed to detect ER and PR expression should be considered while comparing data from independent studies. As well, the existence of mechanisms of post-transcriptional regulation of ER and PR expression [36, 37] may account for discrepancies between mRNA and protein levels. Below, we discuss in detail the variety of strategies used to assess hormone receptor expression across discrete mammary subpopulations in mice and humans, as well as the resulting findings and what they tell us about the role(s) of hormone receptor expressing cells in the mammary epithelium.

**Table 1:** Expression of ER and PR in distinct normal human mammary epithelial subpopulations

Markers	Phenotype	Description	ER expression	PR expression	Detection method
CD10 CD24 CD44 [33]	CD10^−^ CD24^−^ CD44^+^	Stem/Progenitor cells	–	+/−	Semi-quantitative RT-PCR^a^
EpCAM CD49f CD133 MUC1 CD10 THY1 [28]	EpCAM^+^ CD49f ^−^	Mature Luminal Cells	++	++	Long SAGE^b^ and Affimetrix analysis, qRT-PCR^c^
	MUC1^+^CD133^+^				
	CD10^−^ THY1^−^				
	EpCAM^+^ CD49f^+^	Bipotent progenitors	+	++	
	MUC1^−^ CD133^−^				
	CD10^+^ THY1^+^				
	EpCAM^+^ CD49f^+^	Luminal-restricted progenitors	+++	+	
	MUC1^+^ CD133^+^				
	CD10^−^ THY1^−^				
CD31/CD45 (Lin^d^) EpCAM CD49f ALDH ErbB3 [29]	Lin^−^ EpCAM^+^CD49f^−^	Mature Luminal cells	+++	+++	qRT-PCR
	Lin^−^ EpCAM^+^ CD49f^+^ ALDH^+^ ErbB3^+^	Luminal progenitors (High CFC frequency)	++	++	
	Lin^−^ EpCAM^+^ CD49f^+^ ALDH ^-^ ErbB3^+^	Luminal progenitors (Low CFC frequency)	++	++	
CD31/CD45/CD235a (Lin) EpCAM CD49f [25, 26]	Lin^−^ EpCAM^+^ CD49f^−^	Mature Luminal cells	+++	+++	qRT-PCR; ICC^e^ (% of positive cells)
			55 %	71 %	
	Lin^−^ EpCAM^+^ CD49f^+^	Luminal Progenitor Cells	+	+	
			28 %	2 %	
	Lin^−^ EpCAM^−^ CD49f^High^	MaSC-enriched Basal cells	+/−	+	
			0.2 %	0.0 %	
ALDH [27]	ALDH^+^	Stem/progenitor cells	0 %	ND^f^	ICC; Flow cytometry (% of positive cells)

^a^ *RT-PCR* reverse transcription polymerase chain reaction

^b^ *LongSAGE* long serial analysis of gene expression

^c^ *qRT-PCR* quantitative Real-Time PCR

^d^ *Lin* lineage

^e^ *ICC* immunocytochemistry

^f^ *ND* not determined

**Table 2:** Expression of ER and PR in distinct normal mouse mammary epithelial subpopulations

Markers	Phenotype	Description	ER expression	PR expression	Detection method
CD24 Sca-1 Prominin-1 [30, 32]	CD24^Low^	myoepithelial Cells/myoepithelial Cells	+ 0–1 %	+	qRT-PCR; IF^a^ (% of positive cells)
	CD24^High^ Sca-1^−^/Prominin-1^−^	Luminal cells (High CFC frequency)	+ 1–5 %	+	
	CD24^High^ Sca-1^+^/Prominin-1^+^	Luminal cells (Low CFC frequency)	+++ 80–90 %	+++	
CD45/CD31 (Lin) CD24 CD49f/CD29 [34]	Lin^−^ CD24^+^ CD29 ^Low^/CD49f ^Low^	Luminal cells	+++ 37 %	40 %	qRT-PCR; ICC (% of positive cells)
	Lin^−^ CD24^+^ CD29 ^High^/CD49f ^High^	Stem cells	+ <0.01 %	<0.01 %	
CD45/CD31 (Lin) CD24 CD29 CD61 [35, 26]	Lin^−^ CD24^+^ CD29 ^Low^ CD61^−^	Luminal cells (Low CFC frequency)	+++ 40 %	+++	qRT-PCR; ICC (% of positive cells)
	Lin^−^ CD24^+^ CD29 ^Low^ CD61^+^	Luminal cells (High CFC frequency)	+ 6 %	+	
CD45 CD49f CD24 Sca-1 c-Kit [31]	CD45^−^ CD24^+/High^ Sca-1^−^ c-Kit^+^	Sca-1^−^ Luminal cells (High CFC frequency)	+ 0 %	ND^b^	qRT-PCR; IF (% of positive cells)
	CD45^−^ CD24^+/High^ Sca-1^−^ c-Kit^−^	Sca-1^−^ Luminal cells (Low CFC frequency)	+++ 100 %	ND	
	CD45^−^ CD24^+/High^ Sca-1^+^ c-Kit^+^	Sca-1^+^ Luminal cells (High CFC frequency)	+++ 100 %	ND	
	CD45^−^ CD24^+/High^ Sca-1^+^ c-Kit^−^	Sca-1^+^ Luminal cells (Low CFC frequency)	+++ 100 %	ND	
	CD45^−^ CD24^+/Low^ CD49f ^Low^ Sca-1^−^c-Kit^−^	Myoepithelial cells	+ 0 %	ND	
	CD45^−^ CD24^+/Low^ CD49f ^High^ Sca-1^−^c-Kit^−^	Basal cells (High MRU frequency)	+ 0 %	ND	
	CD45^−^ CD24^+/Low^ CD49f ^Low^ Sca-1^−^c-Kit^+^	Basal cells (Low MRU frequency)	++ 0 %	ND	
CD45/CD31/TER119/ /BP-1 (Lin) EpCAM CD49f CD49b Sca-1 [29]	Lin^−^ EpCAM^High^ CD49f^Low^ Sca-1^−^ CD49b^+^	Luminal cells (High CFC frequency)	+ 15–25 %	+	qRT-PCR; ICC (% of positive cells)
	Lin^−^ EpCAM^High^ CD49f^Low^ Sca-1^+^ CD49b^+^	Luminal cells (Low CFC frequency)	+++ 60–90 %	+++	
	Lin^−^ EpCAM^High^ CD49f^Low^ Sca-1^+^ CD49b^−^	Luminal cells (Very low CFC frequency)	+++ 80–90 %	+++	

^a^ *IF* immunofluorescence

^b^ *ND* not determined

## Hormone Receptor Expression in Functionally Distinct Human Mammary Epithelial Cell Populations

As they rarely co-localise with proliferation markers in the normal breast [20] and are positive for cycle inhibitory factors [38, 39], ER-expressing cells have been historically considered terminally differentiated mammary cells. ER-positive cells are distributed through the breast luminal epithelium, often found in close proximity to ER-negative proliferating cells, supporting the model of an indirect response of proliferating cells to oestrogen [20, 40]. Poorly developed lobules with the least complex architecture and the highest proliferative index seem to be the sites enriched for progenitor cells [41] as well as ER- and PR-positive cells [41, 40].

Despite the typical lack of co-localisation of ER staining and proliferative markers, slowly-dividing cells have been identified nonetheless among ER- and PR-positive cells in human mammary tissue implanted in mice [42]. More recently, it has been shown that a population of dual p27/ER-positive cells has progenitor characteristics and proliferates in response to oestrogen stimulation [43]. The extent to which hormone receptor-positive cells overlap with progenitor cells remains difficult to assess in situ, however, and a number of flow sorting-based approaches have been used to isolate breast epithelial stem, progenitor and differentiated subpopulations and analyse them for ER and PR expression (Table 1).

CD44^High^ CD24^Low^ cells have been reported as breast cancer stem cells [44] but normal mammary cells with this phenotype also have properties of stem/progenitor cells [45]. The CD44^High^ CD24^Low^ phenotype within the (EpCAM^High^ CD49f^+^) luminal cell population from normal human breast selects for ER-negative cells and enriches for luminal progenitors [46]. Interestingly although CD44^+^ epithelial cells from normal breast tissue lack ER and PR [33], they express androgen receptor (AR) [43].

Ginestier et al. reported that high aldehyde dehydrogenase ALDH1 activity is associated with stem\progenitor cell properties in human mammary epithelial cells (HMECs) [47]. In situ analysis of ALDH1 and ER expression showed that cells expressing ALDH1 typically lack expression of ER [48], but ER-positive cells are often adjacent to ALDH1-positive cells [27], suggesting a close hierarchical relationship and/or a paracrine interaction between these two cell types in vivo and consistent with an ER-negative progenitor-like phenotype for ALDH-positive cells.

Based on CD49f and EpCAM expression, Lim et al. defined three mammary epithelial subsets, a basal MaSC-enriched (CD49f^High^ EpCAM^−^), a luminal progenitor (CD49f^+^ EpCAM^+^) and a mature luminal cell (CD49f^−^ EpCAM^+^) population [25, 26]. Both *ESR1* and *PGR* transcripts were preferentially expressed in mature luminal cells rather than luminal progenitor and MaSC-enriched fractions [26]. Consistent with this, most of ER- and PR-positive cells were found in the mature luminal subset, while cells of the MaSC-enriched population did not express these receptors [25]. As predicted from expression of c-Kit in the luminal progenitors and ER in the mature luminal cells in cell separation experiments [26, 25], in situ analysis of c-Kit and ER [49, 50] or PR [50] demonstrated mutually exclusive staining in the breast epithelium.

Using a larger panel of markers, four distinct mammary cell populations, reported as bipotent CFC-enriched (EpCAM^+^ CD49f^+^ MUC1^−^ CD133^−^ CD10^+^ THY1^+^), luminal-restricted CFC-enriched (EpCAM^+^ CD49f^+^ MUC1^+^ CD133^+^ CD10^−^ THY1^−^), mature myoepithelial (EpCAM^+^ CD49f^−^ MUC1^−^ CD133^−^ CD10^+^ THY1^+^) and mature luminal (EpCAM^+^ CD49f^−^ MUC1^+^ CD133^+^ CD10^−^ THY1^−^) cells can be resolved [28, 51, 52]. Expression of *ESR1* mRNA is very heterogeneous across these populations, with the lowest levels in the bipotent CFC-enriched fraction and the highest in the luminal-restricted CFC-enriched fraction. Conversely, luminal-restricted CFC-enriched cells exhibit lower *PGR* mRNA levels compared to bipotent CFC-enriched cells [28]. A similar ER and PR distribution with only a partial overlap between them across these mammary cell subsets has been documented by another study [53], raising the possibility that regulation of PR expression by ER may be cell-context dependent.

Shehata et al. reported that differential ALDH activity and ERBB3 expression can further fractionate the luminal progenitor (EpCAM^+^ CD49f^+^) population into three subpopulations corresponding to undifferentiated highly clonogenic ALDH+ ERBB3^+^ (ALDH+) cells, and relatively differentiated less clonogenic ALDH- ERBB3^+^ (ALDH-) and ALDH- ERBB3^−^ (ERBB3-) cells [29]. All the progenitor subpopulations expressed lower levels of *ESR1* transcript compared to mature luminal (EpCAM^+^ CD49f^−^) cells, whilst no difference in *ESR1* expression was found among the distinct progenitor subsets. All types of luminal progenitors displayed multi-lineage potential when assayed using reconstitution assays, albeit with different frequencies.

There is, therefore, a wealth of data from the human supporting the model that mature luminal cells primarily express ER and PR and mediate the proliferative effects of steroid hormones by paracrine signalling. However, a few studies have indicated an enrichment of PR or ER in the progenitor population [28, 53]. Furthermore, isolation of ER-positive cells from normal breast tissue using a fluorescent reporter downstream of estrogen responsive elements demonstrated that a subset of these ER-positive cells is highly clonogenic in vitro and able to generate progeny restricted to the luminal lineage, including both ER-positve and ER-negative cells, in vivo [27]. A caveat to these studies is the use of cells which have undergone a period in culture, rather than being freshly isolated. One could argue, however, that it is exactly this approach which allows rare ER positive progenitors to expand and be enriched for subsequent analysis. Nevertheless, although there is proportion of cells with proliferative potential within the hormone receptor-positive compartment, this cell population appears to primarily promote proliferation in the mammary gland by paracrine stimulation of cells lacking ER and PR.

## Hormone Receptor Expression in Functionally Distinct Mouse Mammary Epithelial Cell Populations

Given its intracellular localization, ER is not currently an antigen amenable to simple routine staining procedures for FACS sorting and functional analysis of ER-positive cells. As an alternative, three main strategies based on expression of cell surface molecules have been developed for the prospective isolation of ER-positive cells from the mouse mammary epithelium (Table 2).

By combining CD24, a marker that allows separation of luminal (CD24^High^) and basal (CD24^Low^) cells [54], with Sca-1 or Prominin-1 staining, our own laboratory demonstrated that luminal cells can be divided in two populations, CD24^High^ Prominin-1^+^/Sca-1^+^ and CD24^High^ Prominin-1^−^/Sca-1^−^, which are enriched in ER-positive and ER-negative cells, respectively [32]. Most of mammary CFCs have a CD24^High^ Sca-1^−^ phenotype, whilst only a small proportion of CFC is comprised within the CD24^High^ Sca-1^+^ population. Both luminal populations have much lower MaSC activity than the CD24^Low^ basal cells, with the ER-positive population having the lowest MRU content [30, 32]. However, the in vivo differentiation potential of the rare luminal transplantable cells is comparable to that of basal MaSCs [32]. Later, we demonstrated that regardless of the ER status, expression of the tyrosine receptor c-Kit positively correlates with progenitor activity in the mouse mammary epithelium [31]. Luminal ER-negative cells express high levels of c-Kit and the few c-Kit^+^ cells within the luminal Sca-1^+^ are highly clonogenic ER-positive cells. On the other hand, the small proportion of luminal Sca-1^−^ cells negative for c-Kit are ER-positive and lack the ability of forming colonies in vitro. In both the ER-positive and ER-negative luminal compartment c-Kit expression is associated with a higher number of MRUs compared to c-Kit negative cells. Together, these results demonstrated that ER expression is mostly dissociated from MaSC and CFC activity but a small population of ER-positive cells with progenitor characteristics exist and can be purified by using a specific set of cell surface markers.

In parallel, other studies have shown that only very few cells in the basal MaSCs (CD29^High^/CD49f^High^ CD24^+^) population express detectable levels of ER and PR, with the great majority of ER- and PR-positive cells being luminal (CD29^Low^/CD49f^Low^ CD24^+^) cells [34]. By using CD61 as additional marker, a highly clonogenic luminal population (CD29 ^Low^ CD24^+^ CD61^+^) can be resolved from a mature luminal cell population (CD29^Low^ CD24^+^ CD61^−^). Most of CD61^+^ luminal cells do not express ER, whereas a high proportion of cells within the CD61^−^ fraction are ER-positive [35].

A third marker used for positive selection of mammary luminal progenitors is CD49b [29]. Whereas the luminal Sca-1^−^ population consisted of mainly CD49b^+^ cells, only a subset of Sca-1^+^ cells exhibited a CD49b^+^ phenotype. As result, using this marker set the luminal epithelium can be divided into a non-clonogenic ER-positive (Sca1^+^ CD49b^−^) population along with a large ER-negative (Sca1^−^ CD49b^+^) and a small ER-positive (Sca1^+^ CD49b^+^) clonogenic cell population. Sca1^+^CD49b^+^ cells differed from Sca1^−^CD49b+ cells for their cloning efficiency and for the morphology of the resulting colonies, indicating that ER-negative and ER-positive progenitors may have distinct functional properties. However, multiple lineage markers could be found in the colonies derived from both cell types. As with our own studies, this work demonstrated that MRUs are present within both the luminal progenitor cell populations, albeit at extremely low frequencies, but the resulting outgrowths were morphologically normal and contain all the mammary cell lineages like those derived from basal cells.

## Lineage Tracing and Hormone Receptor Expression

A major caveat to both in vitro and in vivo proliferation/differentiation assays on purified cell populations is that the differentiation potential and cell fate of progenitors may be altered outside the normal tissue environment. Fortunately, mouse models offer powerful tools for in vivo lineage tracing [6, 8]. To date, however, no lineage tracing study has specifically addressed the developmental fate of ER-negative and ER-positive cells but recent experiments have provided some hints regarding the hormone receptor status of mammary precursors identified in situ.

Lineage tracing of cells expressing Notch-3, revealed that Notch-3 marks rare and mostly quiescent cells that are recruited during mammary gland development to generate luminal cells, both ER- negative and ER- positive [16]. Notch-3-positive precursors are heterogeneous for ER expression [16], and, of note, Notch-3-derived clones are composed exclusively by either ER-positive or ER-negative cells [55].

Tracing experiments using the promoter of another member of the Notch family, Notch-1, showed that in postnatal glands Notch-1-positive precursor cells as well as their descendants are luminal ER- and PR- negative cells [55]. Given their massive expansion during pregnancy along with the ability to generate alveolar secretory cells and survive upon successive involutions, Notch-1-labelled cells are reminiscent of the progenitor population, known as parity-identified mammary epithelial cells (PI-MECs) [56].

By using a lineage tracing strategy, Chang et al. isolated PI-MECs from involuting glands after a single pregnancy and found that these cells are luminal ER- and PR-negative cells and express high levels of alveolar cell markers such as ElF5 and β-Casein [14]. In contrast to the broad differentiation potential shown by PI-MECs in transplantation assays [56], the lineage potential of PI-MECs in intact mammary glands is almost entirely restricted to luminal steroid receptor-negative alveolar cells. During pregnancy, most of the alveolar luminal cells, which are ER-negative, are generated by PI-MECs [14] but the few luminal ER-positive or basal cells within the developing alveoli do not seem to derive from PI-MECs. A complementary picture has been observed in Notch-2-lineage tracing experiments, which have recently revealed the existence of two rare cell types, namely L (large) and S (small) cells as descendants of Notch-2-positive precursors. In particular, during pregnancy, a small number of L cells are present in the luminal layer of each alveolus whilst the majority of alveolar cells do not arise from Notch-2-positive progenitors [15]. Such peculiar scenario warrants the evaluation of steroid-receptor expression in L cells and in their Notch-2 expressing precursors.

## Features of Hormone-Receptor-Positive Luminal Cells

Several lines of evidence highlighted that ER marks a cell population specialized in sensing levels of multiple systemic hormones and converting them into local paracrine signals. Indeed, in addition to elevated levels of ER, this cell population exhibits high expression of PR and Prolactin receptor (Prlr) [26, 30–32, 57], as well as of major paracrine mediators of hormonal signalling, such as AREG [30] and RANKL [26, 57].

Experiments in which ER-null and wild-type mammary cells were transplanted either independently or as mixed populations demonstrated that ER is required for mammary morphogenesis by acting through a paracrine mechanism, as the ER-null cells could contribute to outgrowths only in the presence of wild-type epithelium [58]. It still remains to be established whether the ER-null epithelium failed to develop because of a functional defect in the hormone-sensing cell population or because the genetic ablation of ER resulted in the ablation of the hormone-sensing compartment itself.

A detailed description of the various molecular pathways activated by steroid hormones in cells expressing their cognate receptors is beyond the scope of this review. In brief, both ER and PR mainly localize to the nucleus where they function as gene transcription regulators [5]. However, in addition to genomic effects, activated ER and PR can also induce more rapid responses by initiating cytoplasmic signalling cascades via non-transcriptional mechanisms [5].

Other than ER and PR, only a relative small number of transcription factors, including Foxa1 [26, 29, 30], Msx2 [30], Myb [26, 30], Tbx3 [26], have been reported as specifically expressed in the hormone-sensing cell compartment. Co-expression between ER and the transcription factor GATA-3 has been observed, but mainly in the differentiated luminal compartment [35]. Most of these transcription factors are functionally connected to ER [59, 60], indicating that the ER transcriptional network is likely to be crucial in determining the fate and maintaining the identity of this cell population.

Interestingly, the gene profile of ER-positive luminal cells, isolated from the mammary gland of virgin mice, does not mirror an ‘oestrogen-responsive’ gene signature, defined as the gene sets reported to be up-regulated by oestrogen in normal and tumour mammary cells [30]. This may due to confounding direct and paracrine effects when mixed cell populations are used for the determination of oestrogen-induced genes. Alternatively, it may reflect an intrinsic stable gene expression profile, characteristic of a largely differentiated population, which undergoes subtle undetectable changes in response to relative small fluctuations of hormones in resting virgin glands.

In contrast, luminal cells lacking hormone receptors preferentially express the alveolar lineage-specific transcription factor Elf5 [26, 29, 30, 57, 61] and Lmo4 [26, 29, 30] as well as genes for milk proteins, even in virgin mammary glands [57, 29, 30, 32], suggesting a specific role for these cells as precursors of alveolar secretory cells. Conversely, ER-positive progenitors are likely to be committed along the ductal lineage since they express higher levels of FoxA1 [26, 29, 30], which has been demonstrated to be crucial for ductal but not lobular mammary morphogenesis [59]. Consistent with this, analysis of the in vivo effects of ER genetic ablation showed that ER expression is vital for ductal development but is dispensable for expression of milk protein genes [58].

## Purely a Terminally Differentiated Population?

The molecular analysis of ER-positive and ER-negative cells supports the model of an ER positive terminally differentiated population which drive proliferation of ER-negative cells in a paracrine manner. Lineage tracking suggests ER positive cells can originate from ER negative cells but no groups have specifically tracked ER positive cells to determine whether a subset has progenitor functions and can contribute to the expansion of the ER-positive population itself in vivo. However, current available data indicate that this may indeed be the case.

Transcriptomic analysis of the hormone-sensing population in pregnancy relative to the virgin state has revealed the induction of genes involved in cell cycle and DNA damage repair early during pregnancy, thus unmasking a proliferative potential in this compartment [62]. Indeed, oestrogen-responsive elements have been found in the promoters of several mitotic genes [63]. This is consistent with previous studies indicating that ER- and PR- positive cells proliferate primarily in well-defined windows of normal mammary gland development in vivo. Marked proliferation of hormone receptor-positive cells in situ has been observed during puberty [64] and early pregnancy [65, 24] and in vivo labelling of proliferating cells indicated that a small subset of ER-positive cells corresponds to a slowly dividing cell population in the luminal compartment of mammary epithelium [66–68]. Injection of exogenous steroid hormones stimulates the proliferation of ER- and PR-positive cells [68, 69] while there is a critical role for TGFβ signalling as a negative regulator of ER-positive cell expansion [70]. Although signals that promote proliferation of ER-positive progenitors may be paracrine or juxtacrine – perhaps emanating from more mature ER-positive neighbours – the existence of a cell-autonomous mechanism for stimulating proliferation of ER- and PR-positive human mammary cells by steroid hormones has been proposed by several in vitro studies [43, 71]. One confounding factor, however, in the detection of proliferating ER-positive cells is the rapid ER downregulation that occurs following oestrogen stimulation [72]. A decrease in ER expression before the proliferative response may make difficult to detect ER-positive cycling cells.

The number of ER-positive cells expressing proliferation markers increases after menopause [73, 74]. Since ER expression is inversely correlated to circulating oestrogen levels in mammary epithelial cells [75, 19], it is tempting to speculate that in a low-oestrogen environment the luminal progenitor pool may shift from an ER-negative to an ER-positive state. Notably, hormone-deprivation significantly affects the proliferative capacity of mouse mammary basal stem cells [76] and luminal ER-negative progenitors but not of luminal ER-positive progenitors [29]. However, it is not known whether ER-positive progenitor cells have a similar selective proliferative advantage in human breast after menopause.

## Conclusions and Perspectives

**Fig. 2:**
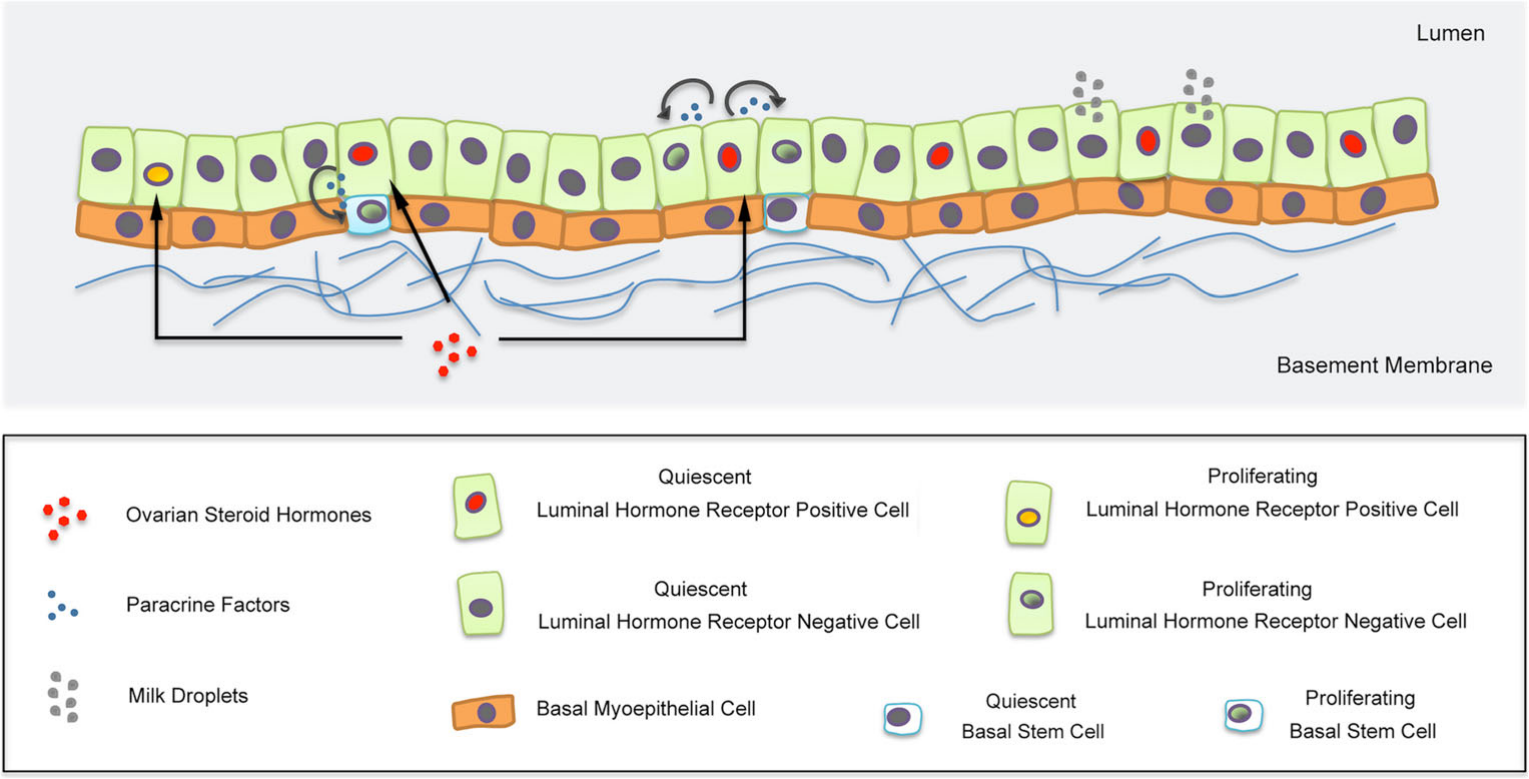
Position and functions of mammary hormone receptor-positive epithelial cells. In response to hormonal stimulation, hormone receptor-positive cells secrete paracrine factors, which induce proliferation of hormone receptor-negative cells. A minor proportion of hormone receptor-positive cells proliferate. Note that there is at present a debate in the field as to whether transplantable MaSCs (MRUs) are a distinct population in the basal epithelium or rather are a subset of myoepithelial cells

Significant advances have been made towards the definition of a high-resolution map of hormone receptor expression in the mammary epithelium. Almost two decades ago the analysis of frequency, distribution and proliferation of hormone receptor-positive cells in situ suggested a paracrine mechanism of hormone action (Fig. 2), but provided limited information about the identity and functional/lineage relationships of these cells or the distinct molecular mechanisms.

More recently, the ability to prospectively isolate and interrogate discrete mammary epithelial cell subpopulations with features of stem, progenitor or mature cells has been crucial for the characterization of the hormone receptor expression pattern along the mammary differentiation hierarchy. Importantly, the identification of cell surface markers to enrich for mammary cells expressing or lacking hormone receptors has allowed the determination of the proliferative capacity and the differentiation potential of these two cell subsets.

Increasing evidence shows that, although a large fraction of hormone receptor-positive cells are mature luminal cells, a minor proportion have properties of progenitors. However, the relationship between the hormone receptor-positive cells characterized as progenitors ex vivo and the rare proliferating hormone receptor-positive cells identified in situ remains to be elucidated. Now, the challenge is to delineate the signalling pathways which specifically regulate the behaviour of these two hormone sensing cell types. A robust strategy for purifying mature and progenitor hormone receptor-positive cells will be necessary for the determination of their gene expression profiles. In addition, investigating the distribution of specific receptor isoforms, co-regulator partners and post-translational modifications may clarify the differential cell response to hormonal stimulation.

An important goal will be the identification of promoters selectively active in the hormone receptor-positive progenitor cells to enable their developmental fate in vivo to be determined through lineage-tracing experiments. Specific promoters could be also used for addressing oncogenic mutations in either differentiated or immature hormone receptor-positive cells, in order to generate novel mouse mammary tumour models, which may more closely resemble specific human breast tumour subtypes and provide important insights into their cellular origin.

Finally, it is interesting to speculate on the profound change seen in the relationship between the majority of proliferating cells and the majority of hormone receptor expressing cells in the normal human breast and in breast cancer. Why do ER-positive cells express proliferative markers in breast cancer but not (in the majority of cells) in the normal breast? Do mature, terminally differentiated cells ER-positive cells revert to a progenitor-like phenotype in breast cancer? Or, is ER-positive breast cancer the result of an uncontrolled expansion of a rare ER-positive progenitor population? Understanding this question will go a long way to understanding the underlying etiology of the most common form of the disease.

## Abbreviations

ALDH: Aldehyde dehydrogenase
AR: Androgen receptor
CFC: Colony-forming cell
ER: Oestrogen receptor
FACS: Fluorescence-activated cell sorting
MaSC: Mammary stem cell
MRU: Mammary repopulating unit
PI-MECs: Parity-identified mammary epithelial cells
PR: Progesterone receptor
Prlr: Prolactin receptor
